# Adequacy of trained assistance, emergency equipment and drugs at emergency calls in the ICU and in remote areas of the hospital

**DOI:** 10.1186/cc14487

**Published:** 2015-03-16

**Authors:** I Kolic, S Unell, A Watts, A Barry, J Short

**Affiliations:** 1 South London Healthcare NHS Trust, London, UK; 2 Lewisham and Greenwich NHS Trust, London, UK

## Introduction

We aimed to identify the adequacy of assistance provided and to assess correct anaesthetic equipment and drug availability at emergency calls made in the ICU and in remote areas of the hospital. Emergency calls often involve managing critically ill patients with the highest mortality results. The importance of a clinical team with the necessary competencies and the right level of resources are paramount.

## Methods

We undertook a prospective survey of all adult patients with emergency calls put out to the anaesthetic team in a London district general hospital over a 6-week period. We performed a snapshot audit of equipment in resuscitation trolleys across each ward and in the radiology department. We compared the data collected on available equipment with the standard set by the Resuscitation Council (UK) Recommended Minimum Equipment Checklist [1]. The survey addressed the availability, clinical competency and appropriate duration of stay of the anaesthetic assistant at the emergency calls. Further qualitative data were collected on the availability of required emergency drugs.

## Results

During the study period 44 emergency calls were attended. Twenty-three (52%) of these calls were in the accident and emergency department, and four (9%) in the ICU. Survey results demonstrated two cases where no anaesthetic assistant arrived at the emergency call put out to them. In cases where timely assistance was available, the assistant did not have the adequate clinical and anaesthetic skills required by the attending physician. In 6% of cases where skilled assistance was required (*n *= 2), it was felt that the assistant did not stay for the clinically required length of time. Emergency drugs required were found to not be available in 11% of cases (*n *= 5) and in 17% of cases (*n *= 6) the necessary emergency equipment was not available. Data were collected on equipment from 17 resuscitation trolleys. The inadequacies identified were the oxygen cylinders were filled less than 75% full in 41% cases (*n *= 7) and end-tidal capnography was identified to be absent.

## Conclusion

Emergency calls require standards to be met involving the competency of responding team members and adequate resources. This leads us to question whether guidelines should exist regarding the clinical competency and timeliness of the assistant available to the physician at emergency calls.
